# Single-inhaler fluticasone furoate/umeclidinium/vilanterol versus fluticasone furoate/vilanterol plus umeclidinium using two inhalers for chronic obstructive pulmonary disease: a randomized non-inferiority study

**DOI:** 10.1186/s12931-018-0724-0

**Published:** 2018-01-25

**Authors:** Peter R. Bremner, Ruby Birk, Noushin Brealey, Afisi S. Ismaila, Chang-Qing Zhu, David A. Lipson

**Affiliations:** 1 University of Notre Dame, Fremantle, WA Australia; 2 GSK, Stockley Park West, Uxbridge, Middlesex, UK; 3 GSK, Collegeville, PA USA; 4 Department of Health Research Methods, Evidence and Impact, McMaster University, Hamilton, ON Canada; 5 GSK, 709 Swedeland Road, UW2531, King of Prussia, PA 19406 USA; 6 Perelman School of Medicine, University of Pennsylvania, Philadelphia, PA USA

**Keywords:** COPD, Exacerbations, FEV_1_, Lung function, Fluticasone furoate/umeclidinium/vilanterol, Randomized controlled trial, Single-inhaler triple therapy

## Abstract

**Background:**

Single-inhaler fluticasone furoate/umeclidinium/vilanterol (FF/UMEC/VI) 100/62.5/25 μg has been shown to improve lung function and health status, and reduce exacerbations, versus budesonide/formoterol in patients with chronic obstructive pulmonary disease (COPD). We evaluated the non-inferiority of single-inhaler FF/UMEC/VI versus FF/VI + UMEC using two inhalers.

**Methods:**

Eligible patients with COPD (aged ≥40 years; ≥1 moderate/severe exacerbation in the 12 months before screening) were randomized (1:1; stratified by the number of long-acting bronchodilators [0, 1 or 2] per day during run-in) to receive 24-week FF/UMEC/VI 100/62.5/25 μg and placebo or FF/VI 100/25 μg + UMEC 62.5 μg; all treatments/placebo were delivered using the ELLIPTA inhaler once-daily in the morning. Primary endpoint: change from baseline in trough forced expiratory volume in 1 s (FEV_1_) at Week 24. The non-inferiority margin for the lower 95% confidence limit was set at − 50 mL.

**Results:**

A total of 1055 patients (844 [80%] of whom were enrolled on combination maintenance therapy) were randomized to receive FF/UMEC/VI (*n* = 527) or FF/VI + UMEC (*n* = 528). Mean change from baseline in trough FEV_1_ at Week 24 was 113 mL (95% CI 91, 135) for FF/UMEC/VI and 95 mL (95% CI 72, 117) for FF/VI + UMEC; the between-treatment difference of 18 mL (95% CI -13, 50) confirmed FF/UMEC/VI’s was considered non-inferior to FF/VI + UMEC. At Week 24, the proportion of responders based on St George’s Respiratory Questionnaire Total score was 50% (FF/UMEC/VI) and 51% (FF/VI + UMEC); the proportion of responders based on the Transitional Dyspnea Index focal score was similar (56% both groups). A similar proportion of patients experienced a moderate/severe exacerbation in the FF/UMEC/VI (24%) and FF/VI + UMEC (27%) groups; the hazard ratio for time to first moderate/severe exacerbation with FF/UMEC/VI versus FF/VI + UMEC was 0.87 (95% CI 0.68, 1.12). The incidence of adverse events was comparable in both groups (48%); the incidence of serious adverse events was 10% (FF/UMEC/VI) and 11% (FF/VI + UMEC).

**Conclusions:**

Single-inhaler triple therapy (FF/UMEC/VI) is non-inferior to two inhalers (FF/VI + UMEC) on trough FEV_1_ change from baseline at 24 weeks. Results were similar on all other measures of efficacy, health-related quality of life, and safety.

**Trial registration:**

GSK study CTT200812; ClinicalTrials.gov NCT02729051 (submitted 31 March 2016).

## Background

The Global Initiative for Chronic Obstructive Lung Disease (GOLD) strategy document recommends escalating to combination triple therapy with a long-acting β_2_-agonist (LABA), a long-acting muscarinic antagonist (LAMA) and an inhaled corticosteroid (ICS) for patients with advanced chronic obstructive pulmonary disease (COPD) and persistent symptoms (GOLD Group D) who experience further symptoms or exacerbations on dual LABA/LAMA or LABA/ICS therapy [1]. Although triple therapy for COPD using multiple inhalers is common in current clinical practice [2, 3], the comparative benefits of COPD treatment regimens using single or multiple inhalers are not well understood.

Triple therapy with a LAMA plus ICS/LABA administered using multiple inhalers has been shown to improve forced expiratory volume in 1 s (FEV_1_) and health status, and reduce exacerbations and rescue medication use, in patients with COPD compared with ICS plus LABA dual therapy or LAMA monotherapy [4–9]. Several recent large randomized controlled trials have also assessed the efficacy and safety of triple ICS/LABA/LAMA therapy using a single fixed-dose combination inhaler for patients with COPD at increased exacerbation risk [10–12].

The FULFIL study demonstrated improvements in trough FEV_1_, health status, and reductions in moderate/severe exacerbation rate, with once-daily, single-inhaler fluticasone furoate/umeclidinium/vilanterol (FF/UMEC/VI) versus twice-daily budesonide/formoterol (FOR) [10]. Similarly, the TRILOGY study showed improvements in lung function and exacerbation frequency with a twice-daily, single-inhaler ICS/LABA/LAMA combination of beclomethasone dipropionate (BDP)/FOR/glycopyrronium bromide (GB) compared with BDP/FOR alone [11]. Furthermore, results from the TRINITY study confirmed that the twice-daily, single-inhaler BDP/FOR/GB combination was non-inferior to twice-daily BDP/FOR plus tiotropium using multiple inhalers on change from baseline in pre-dose FEV_1_ [12].

Given that single-inhaler triple therapy is soon expected to be widely available, the current study was specifically designed to demonstrate the non-inferiority of the only currently available once-daily, single-inhaler triple therapy (FF/UMEC/VI) to an alternative once-daily triple therapy regimen using two inhalers (FF/VI + UMEC) on trough FEV_1_ after 24 weeks of treatment. To our knowledge, this is the first study to specifically evaluate the same individual component molecules administered using either a single inhaler or multiple inhalers.

## Methods

### Study design

**Fig. 1:**
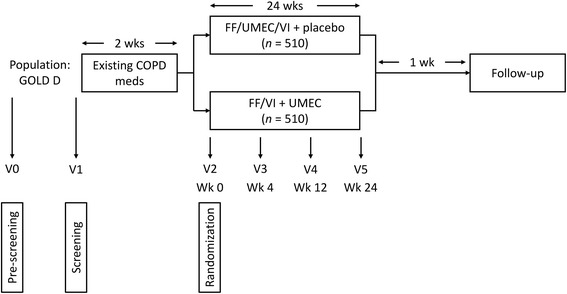
Study design

This was a phase III, 24-week, randomized, double-blind, parallel group, multicenter non-inferiority study (GSK study CTT200812; ClinicalTrials.gov identifier NCT02729051) that assessed the efficacy of once-daily FF/UMEC/VI 100 μg/62.5 μg/25 μg using a single ELLIPTA inhaler versus once-daily FF/VI 100 μg/25 μg plus UMEC 62.5 μg using two ELLIPTA inhalers (Fig. 1). Prior to the beginning of the 24-week treatment period, there was a 2-week run-in period, during which patients continued their existing COPD medications. At randomization following the run-in period, all existing COPD medications were discontinued and patients started their assigned study treatment, with short-acting albuterol/salbutamol provided as rescue medication throughout the study. Study clinic visits occurred at pre-screening (visit 0), screening (visit 1), randomization (Week 0, visit 2), Week 4 (visit 3), Week 12 (visit 4), and Week 24 (visit 5). A safety follow-up telephone contact or clinic visit was conducted a week after study completion, or in the event of an early withdrawal.

### Patients

Patients aged ≥40 years with COPD and who were current/former smokers with a ≥ 10-pack-year smoking history were eligible for enrollment. Other key inclusion criteria included: COPD Assessment Test™ (CAT) score ≥ 10 [13, 14]; a post-albuterol/salbutamol FEV_1_/forced vital capacity ratio < 0.70; and a post-bronchodilator FEV_1_ < 50% of predicted and ≥1 moderate/severe exacerbation in the previous 12 months ***or*** a post-bronchodilator FEV_1_ ≥ 50% to < 80% of predicted and ≥2 moderate exacerbations or ≥1 severe exacerbation requiring hospitalization in the previous 12 months.

Exclusion criteria included: a current diagnosis of asthma (patients with a prior history of asthma were eligible if they had a current diagnosis of COPD that was the primary cause of their respiratory symptoms); α1-antitrypsin deficiency; active tuberculosis; other respiratory disorders that were the primary cause of respiratory symptoms; lung resection surgery in the previous 12 months; risk factors for pneumonia (including immunosuppression and neurological disorders affecting control of the upper airway [e.g. Parkinson’s disease or myasthenia gravis]; pneumonia and/or moderate/severe exacerbation that had not resolved at least 14 days prior to screening; respiratory infections; abnormal findings on chest X-ray; clinically significant comorbidities; unstable liver or cardiac disease; and cancer. Patients with a high risk for pneumonia (e.g. very low body mass index, severely malnourished, or very low FEV_1_) were only to be included at the discretion of the investigator.

All patients provided written, informed consent prior to enrollment.

### Treatments

Eligible patients were randomized (1:1) to receive 24 weeks of FF/UMEC/VI 100 μg/62.5 μg/25 μg in a single inhaler and placebo (second inhaler) or FF/VI 100 μg/25 μg and UMEC 62.5 μg, in separate inhalers; all treatments/placebo were delivered using the ELLIPTA inhaler once daily in the morning. Randomization was stratified by the number of long-acting bronchodilators (0, 1, or 2) per day during the run-in.

### Study assessments

The primary efficacy endpoint was change from baseline in trough FEV_1_ at Week 24. Secondary efficacy endpoints included: proportion of responders based on the St George’s Respiratory Questionnaire (SGRQ) Total score at Week 24; change from baseline in SGRQ Total score at Week 24; proportion of responders based on Transitional Dyspnea Index (TDI) focal score at Week 24; TDI focal score at Week 24; and time to first moderate/severe exacerbation. Safety endpoints included the incidence of adverse events (AEs), serious AEs (SAEs), and AEs of special interest (AESIs).

Spirometry was performed at screening and pre-dose at each scheduled study visit during the treatment period using standardized equipment according to American Thoracic Society–European Respiratory Society guidelines [15]. The SGRQ for COPD patients [16] was completed by patients at randomization and Weeks 12 and 24. The Baseline Dyspnea Index was measured at randomization and the TDI was measured at Weeks 12 and 24; these assessments were completed electronically by patients using self-administered computerized versions. Potential COPD exacerbations were identified based on patient-reported symptoms in an eDiary and confirmed by follow-up with the investigator. Exacerbations were defined as worsening of COPD symptoms that were mild (self-managed by the patient; corticosteroids or antibiotics were not required), moderate (required oral/systemic corticosteroids and/or antibiotics), or severe (required hospitalization). The investigators and their study staff were responsible for detecting, documenting, and reporting AEs at each study visit. The CAT was completed at screening using the eDiary, before any other assessments, to assess eligibility.

### Statistical analyses

Sample size calculations used a one-sided 2.5% significance level and an estimate of residual standard deviation (SD) for trough FEV_1_ at Week 24 of 220 mL (the SD estimate was based on previous phase III studies in patients with COPD). A study with 816 evaluable patients for the primary analysis would have 90% power to determine non-inferiority of FF/UMEC/VI versus FF/VI + UMEC based on trough FEV_1_ at Week 24, when the margin of non-inferiority is 50 mL and the true mean treatment difference is assumed to be 0 mL. It was estimated that ~ 20% of patients who were randomized would either discontinue study treatment or be excluded from the modified per-protocol (mPP) population at Week 24, and so ~ 1020 patients were planned for randomization.

The intent-to-treat (ITT) population included all randomized patients, except those randomized in error. The mPP population included all patients in the ITT population who did not have a protocol deviation affecting efficacy. Data following a severe/moderate COPD exacerbation or pneumonia were excluded from the analysis due to the potential impact of the event or the medications used to treat it. Patients with partial protocol deviations considered to impact efficacy were included in the mPP population but had their data excluded from analyses from the time of deviation onwards.

The primary efficacy endpoint was analyzed for the mPP population using a mixed model repeated measures (MMRM) analysis, including trough FEV_1_ at Weeks 4, 12, and 24. The model included covariates of stratum (number of long-acting bronchodilators per day during the run-in [0/1 or 2]), baseline FEV_1_, visit, center group, treatment, visit by baseline, and visit by treatment interaction. The non-inferiority margin was set at 50 mL (half the minimal clinically important difference [MCID] for trough FEV_1_ in COPD [17]. If the lower bound of the two-sided 95% confidence interval (CI) around the FF/UMEC/VI versus FF/VI + UMEC treatment difference was above − 50 mL, then FF/UMEC/VI was considered non-inferior to FF/VI + UMEC. The MMRM analysis was repeated for the ITT population. All other endpoints were analyzed for the ITT population only.

The proportion of responders based on the SGRQ Total score at Weeks 12 and 24 was analyzed using a generalized linear mixed model, including covariates of baseline SGRQ Total score, treatment group, number of long-acting bronchodilators per day during the run-in (0/1 or 2), geographic region, visit, visit by baseline interaction, and visit by treatment interaction. The number and proportion of responders and non-responders for each treatment at Weeks 12 and 24 was calculated and an odds ratio (OR) for the comparison between FF/UMEC/VI and FF/VI + UMEC with associated 95% CI was provided. The proportion of responders based on the TDI focal score was analyzed in the same way for proportion of responders based on SGRQ Total score. Change from baseline in SGRQ Total score and change from baseline in TDI total score at Weeks 12 and 24 were analyzed separately as described for the primary analysis.

A Cox proportional hazards model was used to compare the time to first moderate/severe COPD exacerbation during 24 weeks of treatment with either FF/UMEC/VI or FF/VI + UMEC. This model used covariates of treatment group, gender, exacerbation history (0, 1, or 2 moderate/severe exacerbations within 12 months of screening), smoking status at screening, number of long-acting bronchodilators per day during the run-in (0/1 or ≥2), geographic region, and baseline percent predicted FEV_1_. A Kaplan–Meier analysis was performed to produce a figure showing Kaplan–Meier survivor functions of the proportion of patients with a first moderate/severe exacerbation over time for each treatment group.

The number and proportion of patients experiencing at least one AE of any type, AEs within each body system, and AEs within each Medical Dictionary for Regulatory Activities preferred term were recorded for each treatment group. Separate summaries were provided for all AEs, drug-related AEs, fatal AEs, non-fatal SAEs, AESIs, and AEs leading to withdrawal. SAEs and deaths were documented in case-narrative format.

## Results

### Patients

A total of 1311 patients were enrolled, of whom 1055 were randomized to receive study treatment (ITT population; FF/UMEC/VI, *n* = 527; FF/VI + UMEC, *n* = 528); 956 patients were included in the mPP population (FF/UMEC/VI, *n* = 478; FF/VI + UMEC, *n* = 478). In the ITT population, 94% of patients completed the study in each treatment group. Patient demographics and clinical characteristics in the ITT population were generally well balanced between the treatment groups, with no significant differences in terms of disease severity, GOLD grade or exacerbation history at baseline (Table 1); current and past medical conditions and the incidence of cardiovascular risk factors at baseline were also similar between the two treatment arms. Patient characteristics in the mPP population were similar (not shown).

**Table 1:** Patient demographics and clinical characteristics (ITT population)

Characteristic	FF/UMEC/VI 100/62.5/25 μg	FF/VI 100/25 μg + UMEC 62.5 μg	Total
	(*N* = 527)	(*N* = 528)	(*N* = 1055)
Age (years), mean (SD)	66.7 (8.5)	65.9 (8.8)	66.3 (8.6)
Female, *n* (%)	136 (26)	134 (25)	270 (26)
Current smoker at screening, *n* (%)	209 (40)	192 (36)	401 (38)
Smoking pack-years, mean (SD)	43.4 (23.9)	44.2 (25.2)	43.8 (24.6)
Current cardiovascular risk factors, *n* (%)	379 (72)	367 (70)	746 (71)
Number of exacerbations in previous 12 months, *n* (%)^a^			
1 moderate/severe	236 (45)	227 (43)	463 (44)
≥ 2 moderate/severe	291 (55)	301 (57)	592 (56)
≥ 2 moderate or ≥1 severe	352 (67)	360 (68)	712 (67)
History of pneumonia, *n* (%)	86 (16)	100 (19)	186 (18)
Screening lung function, mean (SD)	*n* = 515	*n* = 512	*n* = 1027
Post-bronchodilator FEV_1_, mL	1247 (465)	1297 (471)	1272 (469)
Post-bronchodilator FVC, mL	2879 (885)	2896 (849)	2887 (867)
Post-bronchodilator FEV_1_/FVC ratio	0.440 (0.116)	0.455 (0.119)	0.447 (0.118)
Post-bronchodilator percent predicted FEV_1_	44.5 (14.5)	45.5 (14.1)	45.0 (14.3)
Percent reversibility	9.02 (11.22)	8.87 (10.15)^b^	8.95 (10.69)
Number of long-acting bronchodilators per day during the run-in, *n* (%)			
0/1	225 (43)	226 (43)	451 (43)
2	302 (57)	302 (57)	604 (57)
Concomitant COPD medications taken at screening, *n* (%)			
Single-inhaler maintenance bronchodilator	40 (8)	42 (8)	82 (8)
LAMA	32 (6)	35 (7)	67 (6)
LABA	8 (2)	7 (1)	15 (1)
Combination therapy	448 (85)	443 (84)	891 (84)
ICS + LABA+LAMA	198 (38)	193 (37)	391 (37)
ICS + LABA	144 (27)	137 (26)	281 (27)
LABA+LAMA	62 (12)	76 (14)	138 (13)
ICS + LABA+LAMA+ xanthine	29 (6)	25 (5)	54 (5)
ICS + LAMA	7 (1)	9 (2)	16 (2)
LABA+LAMA+xanthine	8 (2)	3 (< 1)	11 (1)
COPD severity at screening			
GOLD grade, n (%)	*n* = 515	*n* = 512	*n* = 1027
1 (mild)	0	1 (< 1)	1 (< 1)
2 (moderate)	174 (34)	189 (37)	363 (35)
3 (severe)	251 (49)	253 (49)	504 (49)
4 (very severe)	90 (17)	69 (13)	159 (15)
Reversible, *n* (%)^c^	n = 515	*n* = 511	*n* = 1026
Yes	73 (14)	74 (14)	147 (14)
GOLD grade/exacerbation history, *n* (%)^a^	*n* = 514	*n* = 512	*n* = 1026
Grade 1/2 with ≥2 moderate or ≥1 severe	173 (34)	190 (37)	363 (35)
Grade 3/4 with < 2 moderate and no severe	171 (33)	164 (32)	335 (33)
Grade 3/4 with ≥2 moderate or ≥1 severe	170 (33)	158 (31)	328 (32)
CAT score, mean (SD)	19.6 (5.8)	20.1 (6.1)	19.9 (6.0)

*CAT* COPD Assessment Test™, *COPD* chronic obstructive pulmonary disease, *FEV*_*1*_ forced expiratory volume in 1 s, *FF* fluticasone furoate, *FVC* forced vital capacity, *GOLD* Global Initiative for Chronic Obstructive Lung Disease, *ICS* inhaled corticosteroid, *ITT* intent-to-treat, *LABA* long-acting β_2_-agonist, *LAMA* long-acting muscarinic antagonist, *SD* standard deviation, *UMEC* umeclidinium, *VI* vilanterol

^a^Moderate exacerbations were defined as exacerbations requiring oral/systemic corticosteroids and/or antibiotics (not involving hospitalization). Severe exacerbations were defined as exacerbations that required in-patient hospitalization

^b^FF/VI + UMEC, *n* = 511

^c^Reversible was an increase in FEV_1_ of ≥12% and ≥200 mL following administration of salbutamol. Not reversible was an increase in FEV_1_ of < 200 or ≥200 mL increase that is < 12% of the pre-salbutamol FEV_1_

### Efficacy

**Fig. 2:**
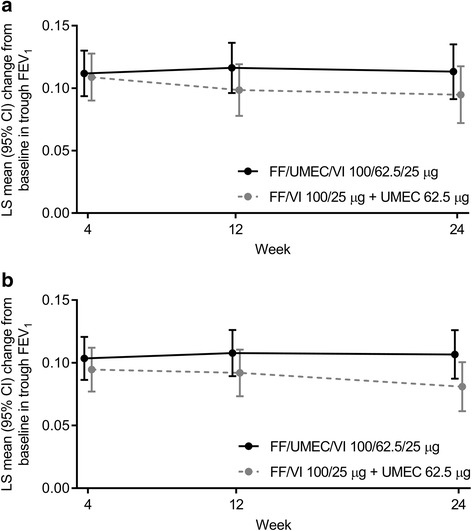
Mean change from baseline in trough FEV_1_ over 24 weeks in **a**: the mPP population and **b**: the ITT population

In the mPP population, the mean change from baseline in trough FEV_1_ at Week 24 was 113 mL (95% CI 91, 135) for FF/UMEC/VI and 95 mL (95% CI 72, 117) for FF/VI + UMEC; the between-treatment difference was 18 mL (95% CI -13, 50) (Fig. 2). As the lower bound of the 95% CI for the comparison was above the predefined non-inferiority margin (− 50 mL), FF/UMEC/VI was considered non-inferior to FF/VI + UMEC. Comparable findings were observed in the ITT population: the mean change from baseline in trough FEV_1_ at Week 24 was 107 mL (95% CI 87, 126) for FF/UMEC/VI and 81 mL (95% CI 61, 100) for FF/VI + UMEC; the between-treatment difference was 26 mL (95% CI -2, 53).

**Fig. 3:**
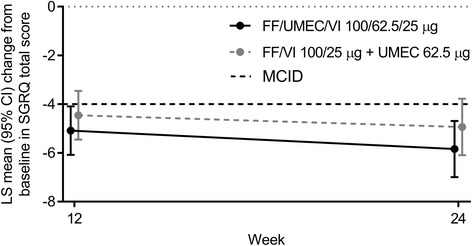
Mean change from baseline in SGRQ Total score over 24 weeks (ITT population)

In the ITT population, the proportion of responders based on the SGRQ Total score at Week 24 was similar in the FF/UMEC/VI group (50%) and FF/VI + UMEC group (51%); the OR of response versus non-response for FF/UMEC/VI versus FF/VI + UMEC was 0.92 (95% CI 0.71, 1.20). The mean change from baseline in SGRQ Total score at Week 24 was − 5.8 (95% CI -7.0, − 4.7) for FF/UMEC/VI and − 4.9 (95% CI -6.1, − 3.8) for FF/VI + UMEC; the between-treatment difference was − 0.9 (95% CI -2.5, 0.7) (Fig. 3). The proportion of responders based on TDI focal score at Week 24 was the same for FF/UMEC/VI and FF/VI + UMEC (56% in each group; OR of response versus non-response for FF/UMEC/VI versus FF/VI + UMEC was 0.95 [95% CI 0.72, 1.25]). The mean TDI focal score at Week 24 was 2.0 (95% CI 1.8, 2.3) for FF/UMEC/VI and 1.9 (95% CI 1.6, 2.1) for FF/VI + UMEC; the between-treatment difference was 0.1 (95% CI -0.2, 0.5).

**Fig. 4:**
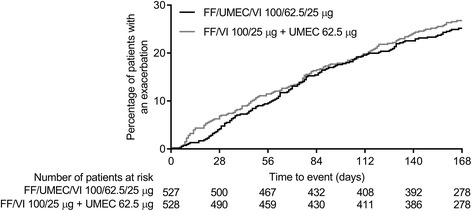
Time to first on-treatment moderate/severe exacerbation over the 24-week treatment period (ITT population)

A similar proportion of patients (ITT population) experienced a moderate/severe exacerbation in the FF/UMEC/VI group (24%) and FF/VI + UMEC group (27%) (Table 2). The hazard ratio for time to first on-treatment moderate/severe exacerbation with FF/UMEC/VI versus FF/VI + UMEC was 0.87 (95% CI 0.68, 1.12) (Fig. 4).

**Table 2:** Summary of on-treatment COPD exacerbations (ITT population)

	F/UMEC/VI 100/62.5/25 μg	FF/VI 100/25 μg + UMEC 62.5 μg
	(*N* = 527)	(*N* = 528)
Patients with a mild, moderate or severe exacerbation, *n* (%)	134 (25)	145 (27)
Mild	8 (2)	5 (< 1)
Moderate	111 (21)	118 (22)
Severe	22 (4)	31 (6)
Moderate/Severe	129 (24)	142 (27)
Number of moderate/severe exacerbations, *n* (%)		
0	398 (76)	386 (73)
1	105 (20)	111 (21)
≥ 2	24 (5)	31 (6)

*COPD* chronic obstructive pulmonary disease, *FF* fluticasone furoate, *ITT* intent-to-treat, *SD* standard deviation, *UMEC* umeclidinium, *VI* vilanterol

### Safety

The proportion of patients who experienced at least one AE was comparable between both treatment groups (48%); the proportion of patients who had at least one SAE was 10% in the FF/UMEC/VI group and 11% in the FF/VI + UMEC group (Table 3). The most frequent AEs were viral upper respiratory tract infection (FF/UMEC/VI, 11%; FF/VI + UMEC, 10%), headache (6% in each group), and COPD (FF/UMEC/VI, 4%; FF/VI + UMEC, 6%). The incidence of AESIs was similar between the treatment groups, including pneumonia (FF/UMEC/VI, 3%; FF/VI + UMEC, 4%) and cardiovascular events (FF/UMEC/VI, 6%; FF/VI + UMEC, 5%) (Table 3).

**Table 3:** Overview of safety findings (ITT population)

Category, *n* (%)	FF/UMEC/VI 100/62.5/25 μg	FF/VI 100/25 μg + UMEC 62.5 μg
	(*N* = 527)	(*N* = 528)
On-treatment AEs	255 (48)	253 (48)
On-treatment drug-related AEs	27 (5)	19 (4)
On-treatment SAEs	52 (10)	57 (11)
On-treatment non-fatal SAEs	50 (9)	54 (10)
On-treatment fatal SAEs	4 (< 1)	4 (< 1)
On-treatment AESIs^a^		
Adrenal suppression	1 (< 1)	0
Anticholinergic syndrome	12 (2)	5 (< 1)
Cardiovascular events	30 (6)	28 (5)
Cardiac arrhythmia	6 (1)	8 (2)
Cardiac failure	7 (1)	7 (1)
Ischemic heart disease	6 (1)	3 (< 1)
Hypertension	10 (2)	13 (2)
CNS hemorrhage/cerebrovascular conditions	3 (< 1)	1 (< 1)
Decreased bone mineral density and associated fractures	5 (< 1)	6 (1)
Hyperglycemia/new-onset diabetes mellitus	7 (1)	6 (1)
Hypersensitivity	7 (1)	9 (2)
LRTI excluding pneumonia	16 (3)	11 (2)
Local steroid effects	12 (2)	14 (3)
Ocular effects	4 (< 1)	5 (< 1)
Pneumonia	14 (3)	21 (4)
Tremor	1 (< 1)	0

*AE* adverse event, *AESI* adverse event of special interest, *CNS* central nervous system, *FF* fluticasone furoate, *ITT* intent-to-treat, *LRTI* lower respiratory tract infection, *SAE* serious adverse event, *SD* standard deviation, *UMEC* umeclidinium, *VI* vilanterol

^a^No events were reported for the asthma/bronchospasm, effects on potassium, gastrointestinal obstruction, or urinary retention AESI groups

## Discussion

This study aimed to evaluate an important clinical outcome in patients with COPD following treatment with the same three individual component molecules administered using either a single inhaler or two inhalers. While this appears to be scientifically self-evident, this was the first study to specifically demonstrate non-inferiority of a single-inhaler triple pharmacologic regimen to the same treatments delivered using multiple inhalers.

This study showed that single-inhaler FF/UMEC/VI 100 μg/62.5 μg/25 μg is non-inferior to FF/VI 100 μg/25 μg plus UMEC 62.5 μg using two inhalers based on change from baseline in trough FEV_1_ at Week 24. The non-inferiority margin for the lower 95% confidence limit was set at − 50 mL, which is half the MCID for trough FEV_1_ in COPD [17]. The proportions of responders based on SGRQ Total score and TDI focal score, and the time to first on-treatment moderate/severe exacerbation, were similar between the treatment groups. As expected, the incidence of AEs, SAEs, and AESIs was comparable for FF/UMEC/VI and FF/VI + UMEC; the incidence of pneumonia AESI was 3% and 4% for FF/UMEC/VI and FF/VI + UMEC, respectively. These results demonstrate that the efficacy and safety of single-inhaler FF/UMEC/VI are similar to the same treatments delivered using two inhalers in patients with COPD. Our findings are supported by previous research, which showed that the systemic exposure to FF, UMEC, and VI following administration as a single-inhaler combination is similar to that observed with the dual therapies FF/VI and UMEC/VI [18].

The mean change from baseline in trough FEV_1_ at Week 24 with FF/VI/UMEC in the ITT population in the current study (107 mL) is lower than the change from baseline reported with FF/UMEC/VI in the ITT population in the FULFIL study at 24 weeks (142 mL) [10]. This likely reflects the fact that 80% of the enrolled population in the current study used dual, triple, or quadruple combination therapies at baseline. Nevertheless, this reduced level of improvement in lung function was associated with a clinically meaningful improvement from baseline in SGRQ Total score at Week 24 of − 5.8 units, which is consistent with the − 6.6-unit change reported with FF/UMEC/VI in the FULFIL study [10]. It should also be noted that the mean improvement from baseline in trough FEV_1_ at Week 24 with FF/VI/UMEC in the ITT population in the current study was 26 mL (95% CI -2, 53) greater than that observed with FF/VI + UMEC using two inhalers. This indicates some potential for greater consistency in the bronchodilator effect for the single-inhaler combination across all patients, compared with the two-inhaler regimen.

Our findings are also in line with a previous 52-week study comparing twice-daily, single-inhaler BDP/FOR/GB triple therapy with twice-daily BDP/FOR plus tiotropium using multiple inhalers, which showed non-inferiority for the single-inhaler formulation based on change from baseline in pre-dose FEV_1_ (between-treatment difference: − 3 mL [95% CI -33, 27]) [12]. The incidence of AEs and SAEs with FF/UMEC/VI observed in this study (48% and 10%, respectively) was higher than the incidences observed with FF/UMEC/VI in the FULFIL trial (39% and 5%, respectively). The current study was designed to recruit a population with higher disease burden and exacerbation risk, compared to the population recruited to the FULFIL trial [10], which likely accounts for the differences seen in adverse event reporting between the two studies. These differences are therefore not considered to be clinically relevant. The incidence of pneumonia AESI in the FF/UMEC/VI group in the current study was relatively low (3%) despite 16% of patients enrolled in this group having a history of pneumonia.

A once-daily, single-inhaler treatment regimen offers a simplified dosing option that may provide a number of benefits to patients with COPD, such as reducing the number of obligatory co-pays in markets where patients are required to subsidize the cost of their medicines. A single-inhaler regimen may also help to ensure that the prescribed combination is delivered consistently and reduce the risk of inhaler errors [19–21]. This may improve patient adherence and outcomes, and reduce associated healthcare costs. However, in the current study, patients in both groups received their assigned study treatment using double-dummy blind inhalers, and so we were unable to directly assess the impact on adherence of a simplified single-inhaler triple therapy regimen versus the same treatments delivered using two inhalers. Nevertheless, as numerical treatment differences in favor of FF/VI/UMEC versus UMEC+FF/VI were consistently observed in the ITT population for both mean change from baseline in FEV_1_ and SGRQ total score, it is possible that a more pragmatic, real-world efficacy study might demonstrate such efficacy benefits.

Our results are specific to FF/UMEC/VI administered using the ELLIPTA inhaler, so similar clinical findings may not be observed when different molecules or inhaler devices are combined. Another study limitation was the lack of a control group receiving dual ICS/LABA or LAMA/LABA therapy, although a dual therapy control group was included in the FULFIL and forthcoming IMPACT trials [10, 22]. A potential study strength was that 80% of enrolled patients continued their existing dual, triple or quadruple combination therapies during the 2-week run-in period, and 67% continued to remain at high risk of exacerbations. This meant that the study population more closely resembled a real-world COPD population who may benefit most from the simplicity afforded by a once-daily, single-inhaler triple therapy regimen.

## Conclusions

This study showed that single-inhaler FF/UMEC/VI 100 μg/62.5 μg/25 μg was non-inferior to FF/VI 100 μg/25 μg plus UMEC 62.5 μg based on change from baseline in trough FEV_1_ at Week 24 in patients with advanced COPD. Our findings confirm that single-inhaler triple therapy with FF/UMEC/VI offers similar efficacy, health-related quality of life, and safety benefits as the same triple therapy administered using two inhalers.

## Abbreviations

AE: Adverse event
AESI: Adverse event of special interest
BDP: Beclomethasone dipropionate
CAT: COPD Assessment Test™
CI: Confidence interval
CNS: Central nervous system
COPD: Chronic obstructive pulmonary disease
FEV_1_: Forced expiratory volume in 1 s
FF: Fluticasone furoate
FOR: Formoterol
FVC: Forced vital capacity
GB: Glycopyrronium bromide
GOLD: Global initiative for chronic obstructive lung disease
ICS: Inhaled corticosteroid
ITT: Intent-to-treat
LABA: Long-acting β_2_-agonist
LAMA: Long-acting muscarinic antagonist
LRTI: Lower respiratory tract infection
MCID: Minimal clinically important difference
MMRM: Mixed model repeated measures
mPP: Modified per-protocol
OR: Odds ratio
SAE: Serious adverse event
SD: Standard deviation
SGRQ: St George’s respiratory questionnaire
TDI: Transitional dyspnea index
UMEC: Umeclidinium
VI: Vilanterol
